# Artemisinin-Naphthoquine Combination (ARCO^®^): An Overview of the Progress

**DOI:** 10.3390/ph3123581

**Published:** 2010-12-14

**Authors:** Francis W. Hombhanje, Qingyun Huang

**Affiliations:** 1 Centre for Health Research, Divine Word University, P O Box 483, Madang 555, Madang Province, Papua New Guinea; 2 Medical Research Institute, Kunming Pharma, West suburb, Xishan Region, Kunming, China; E-Mail: sooenaing@126.com

**Keywords:** artemisinin-naphthoquine combination, ARCO, artemisinin, naphthoquine, *Plasmodium falciparum*

## Abstract

With the rapidly spreading resistance of *Plasmodium falciparum* to available non-artemisinin antimalarial drugs, new and novel pharmaceuticals are needed. ARCO^®^ is a new generation ACT, one of several artemisinin-based combinations developed in China to counter antimalarial drug resistance. ARCO^®^ is a derivative of two independently developed antimalarials, artemisinin and naphthoquine phosphate, which were combined to form the artemisinin-naphthoquine combination. Both artemisinin and naphthoquine drugs have proven to be efficacious, safe and well tolerated as monotherapies. The artemisinin-naphthoquine combination offers a novel advantage over existing ACTs: it can be administered as a single oral dose (or a 1-day treatment). Several therapeutic studies conducted recently indicate that a single oral dose administration of artemisinin-naphthoquine combination is equally effective and safe as the 3-day treatment with artemether-lumefantrine combination and other existing ACTs. This would make ARCO^®^ the next generation ACT for the treatment of uncomplicated falciparum malaria.

## 1. Introduction

Malaria infections, particularly due to *Plasmodium falciparum*, have been on the rise in the past decade, mainly because this species of malaria parasite have developed multiple strategies to deal with the parasiticidal actions of available non-artemisinin antimalarial drugs [1,2,3]. Declining efficacy of artemisinins and/or artemisinin-based combinations (ACTs) is being increasingly observed, particularly along Thai-Cambodia border [4]. These observations pose a serious threat to combination therapies that include artemisinins. Therefore, not only are new pharmaceuticals needed, but new generation pharmaceuticals with novel parasiticidal activities are necessary to deal with the life-threatening situations posed by resistant falciparum parasite pathogen. Several novel drug candidates of synthetic and natural product origin, as well as their combination therapies, have been evaluated that seems to exhibit novel anti-parasite activities, particularly against the multi-drug resistant strains of the *Plasmodium falciparum* species [5]. The primary objective of malaria therapy is to save life by reducing the risks of serious complication and/or severe disease, which ultimately leads to death. This can be achieved by rapidly reducing the total circulating young parasite biomass, thus preventing them from further development to mature stages which are often implicated in severe disease processes and/or complications [6,7,8]. Artemisinin and its derivatives are of special value in this regard, as they achieve a faster reduction in parasitaemia biomass by acting principally on the circulating young parasites [9,10,11,12,13]. Artemisinin-based combination therapies (ACTs) represent a promising approach to dealing with drug-resistant malaria rather than monotherapies. Several ACTs are currently available, including artemether-lumefantrine (AL), dihydroartemisinin-piperaquine (DHA-PPQ), artesunate-amodiaquine (AS + AQ), artesunate-mefloquine (AS + MQ), and now artemisinin-naphthoquine (ARCO^®^). The later combination is of particular interest because, unlike other artemisinin-based combinations which require a 3-day regimen, ARCO^®^ requires either a single dose treatment or a two-dose treatment over 24-hrs (1-day treatment). The effectiveness of this very short therapeutic regimen of ARCO^®^ against uncomplicated falciparum malaria has now been validated by several clinical studies within and outside China [14,15,16,17,18,19]. We provide an overview of ARCO^®^ as the next generation ACT for the treatment of uncomplicated falciparum malaria.

## 2. A Literature Overview

### 2.1. General Aspects

**Figure 1:**
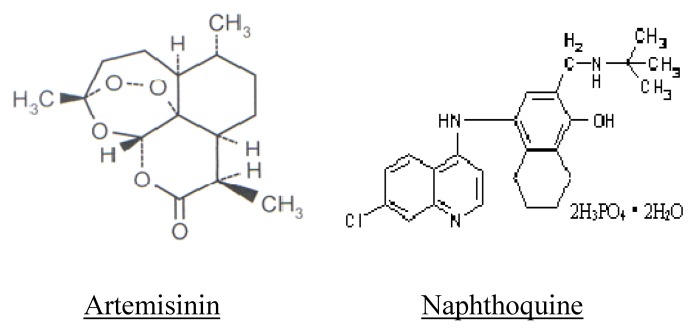
Chemical structure of artemisinin and naphthoquine.

ARCO^®^ is a new generation ACT developed by the Chinese Academy of Military Medical Sciences (AMMS) in the early 1990s. It is a product of the combination of two independently developed antimalarials, artemisinin and naphthoquine (Figure 1). Both drugs have shown to be safe, efficacious, and well tolerated as monotherapies [20,21,22,23]. Artemisinin (a sesquiterpene lactone) is the principal antimalarial compound isolated from the Chinese medicinal herb *Qing hao* (*Artemisia annua*) in the 1970s. Because of its poor water solubility, and hence its poor oral bioavailability, until now it has been paid less attention than its derivatives (artemether, artesunate, dihydroartemisinin, *etc.*) as a potent antimalarial, and therefore artemisinin has not been widely deployed for malaria monotherapy. Naphthoquine is another antimalarial compound developed in China in the late 1980s. It belongs to the 4-aminoquinoline family [15]. The drug (naphthoquine) has never been deployed widely for malaria therapy outside China. Nevertheless, both drugs have demonstrated fairly good pharmacological and anti-parasitological properties as individual antimalarial drugs [24,25,26,27]. The main disadvantages of artemisinin and naphthoquine as monotherapies for malaria infections have been, for artemisinin, a very short circulating half life as a result of rapid elimination, such that effective concentration levels might not be sustained to ensure complete elimination of blood parasites over several asexual cycles. For naphthoquine, the main disadvantage has been the slowness in the onset of the parasiticidal action following therapy administration. The slowness in the onset of action would create a time-window of opportunity for young circulating parasites to escape into the central intra-vascular compartment. The escaped parasites are more likely to avoid the parasiticidal action(s) of the drug. It becomes apparent therefore, that co-formulation should hypothetically overcome the inherent disadvantages of the individual drugs. 

### 2.2. Co-Formulation

The concept of combination therapy is based on the synergistic or additive potential of two or more drugs. Apart from improving therapeutic efficacy, combinations may also delay the development of drug resistance to the individual components of the combination. The rational for artemisinin-naphthoquine combination is for artemisinin component to provide the rapid killing of parasites and accelerate therapeutic response. Its rapid action may also prevent early treatment failure and/or complications by greatly reducing the circulating parasite biomass in a shorter time. The benefits of adding naphthoquine is to prevent recrudescence by killing residual parasites through its extended half-life in the body. This action may also reduce the chance of resistant mutants surviving and propagating. Overall, artemisinin and naphthoquine will complement each other such that the delay time for parasiticidal action of naphthoquine is covered by artemisinin’s immediate onset of parasiticidal action, and the short circulating half life of artemisinin would be extended over several days (covering several asexual cycles) by the long circulating half life of naphthoquine to eliminate any circulating residual parasites. This is the most novel of the drug combinations because the drugs in this combination therapy have not been previously used as monotherapies so as to condition parasites. However, when two drugs are combined into one single tablet, it becomes a new drug entity with its own challenges in regards to safety issues [28,29].

The optimal proportion of naphthoquine phosphate and artemisinin in the combined tablet was determined after a series of painstaking studies [14,15]. The appropriate proportions of naphthoquine and artemisinin that provided optimal efficacy and safety in a single dose were 400 mg and 1,000 mg, respectively. This means, one combined tablet would contain 50 mg naphthoquine base (78.3 mg of naphthoquine phosphate) and 125 mg of artemisinin in a ratio of 1:2.5, and the appropriate dosage regimen would only be one dose. The fixed-dose combination tablets are manufactured, registered, and marketed as ARCO (ARCO^®^) by Kunming Pharmaceutical Corporation (KPC) of the People’s Republic of China. 

### 2.3. Therapeutic Dosage Regimen of ARCO®

The recommended dosage for adult population (age > 16 years) for uncomplicated malaria is a single dose of eight tablets (total dose 1,000 mg artemisinin /400 mg naphthoquine). For children, it is recommended that it be adjusted on a body-weight basis (25 mg artemisinin/10 mg naphthoquine). For younger children, including infants, tablets should be crushed before administration. The manufacturer’s current recommendation is that all medications are to be taken before meals or after meals (~2 h post-prandial) but not with a meal. 

## 3. Clinical and Therapeutic Assessment of ARCO^®^

### 3.1. General Overview

The progress of ARCO^®^ from its development to clinical assessment in general human populations has moved forward quickly. In this section, we present some of the progress that have taken place and provide an overview of recent findings from several clinical trials in the context of the potential applicability of ARCO^®^ in malaria therapy. 

**Figure 2:**
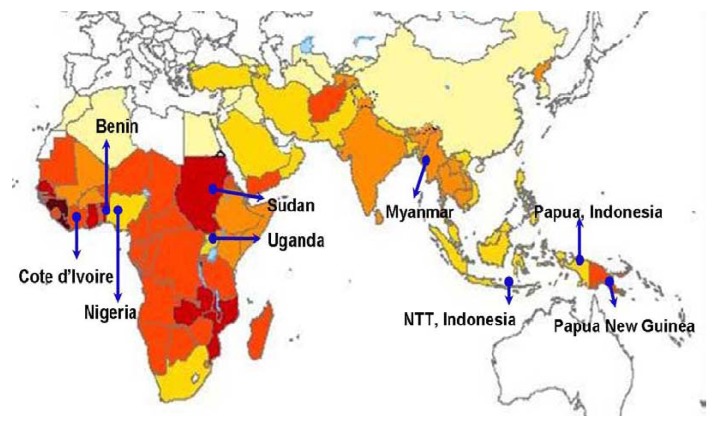
World map indicating countries where ARCO^®^ studies were conducted.

The preliminary clinical assessment undertaken with the single dose administration of ARCO^®^ tablets in China demonstrated high level of efficacy (cure rates of greater than 95%), safety, and tolerability in the adult populations with falciparum and vivax malaria [14,15,16]. Encouraged by these preliminary findings, further efficacy and safety evaluation of ARCO^®^ was undertaken outside China in eight (n = 8) different malaria-endemic countries involving almost 2,000 participants (1,127 adults, 385 children, and 293 adults and children combined), in the Asia-Pacific and African regions (Figure 2). Most of these clinical studies were industry-sponsored, but were conducted independently at different sites. Almost all data for this review was obtained from the industry’s archived files and the review was undertaken objectively and independently. The abstracted profiles of different studies are presented below: 

(i) *ARCO^®^ vs. chloroquine and sulphadoxine-pyrimethamine (SP) combination*. This trial was conducted in an adult population with uncomplicated falciparum malaria infections in Papua New Guinea (Melanesian-Western Pacific). In this setting the ARCO^®^ tablets were administered as a single dose versus chloroquine once a day for three days with a single dose of SP at the start of therapy. The therapeutic responses were monitored for 28 days. Although the two treatments provided relatively comparable cure rates, ARCO^®^ treatment was superior in rate of clearing parasitaemia [19]. 

(ii) *ARCO^®^ vs. dihydroartemisinin-piperaquine* (DHA-PPQ). This study was conducted in Indonesia in an adult population with uncomplicated falciparum malaria, vivax malaria, and in co-infection of falciparum-vivax malaria. In this study, a single dose (8 tablets) of ARCO^®^ versus 3-days (once/day for 3 days) of DHA-PPQ combination tablets was investigated. The therapeutic responses were monitored for 28 and 42 days. Both treatments provided comparable PCR corrected cure rates for *Plasmodium falciparum* (ARCO^®^ 99% *vs.* DHA-PPQ 97%), *Plasmodium vivax* (ARCO^®^ 99% *vs.* DHA-PPQ 97%), and mixed infection of *Plasmodium falciparum* and *Plasmodium vivax* (ARCO^®^ 79% *vs.* DHA-PPQ 97%) malarias at day 42. There was no statistically significant difference in parasite clearance times (ARCO^®^ 28 ± 11.7 *vs*. 26 ± 12.2 DHA-PPQ) for both treatments; however, response to ARCO^®^ treatment was low in mixed infection [source: KPC archived data]. 

(iii) *ARCO^®^ vs. artemether-lumefantrine* (AL). Two of these studies were conducted in children populations of Nigeria and Uganda, and one in an adult population with uncomplicated falciparum malaria in Uganda. For the children, the number of ARCO^®^ tablets administered was based on the bodyweight (25/10 mg/kg artemisinin-naphthoquine combination) and for the adult population ARCO^®^ tablets were administered as a single dose of eight tablets. The therapeutic responses were monitored for 28 days in the Nigerian study and 42 days in the Ugandan study. There was no significant difference in the efficacy and safety profiles in children in the two studies between the single dose ARCO^®^ treatment and 6-dose regimen of AL at day 28 and day 42, respectively. Similar observations were made between the two treatments in the adult population in the Ugandan study at day 28 [Source: KPC archived data].

(iv) *ARCO^®^ vs artemether-lumefantrine vs artesunate-amodiaquine* (three-arm study). This study was conducted in Nigeria in mixed population (children + adults) with uncomplicated falciparum malaria. ARCO^®^ tablets were administered according to the dosing schedule described above. For AL, tablets were administered twice a day for three days based on bodyweight for children and predetermined number of tablets for adults while for AA, once a day treatment for 3 days based on bodyweight for children and predetermined number of combination tablets for adults. The therapeutic responses were monitored for 28 days. The study concluded that ARCO^®^ and AA treatments were marginally better than artemether-lumefantrine in these settings [Source: KPC archived data]. 

(v) *ARCO^®^ (2x/day in divided dosage) vs. artemether-lumefantrine* (*AL)*. This study was undertaken in Ivory Cost, West Africa, in a mixed population (children and adults) with uncomplicated falciparum malaria. Drugs were administered as described above. The therapeutic responses were monitored for 28 days. There was no significant difference in the efficacy and safety of 1-day (2x/day in divided dosage) treatment with ARCO^®^ and 3-day with AL (cure rate: ARCO^®^ 100% *vs.* 98% AL) [18].

(vi) *ARCO^®^ (single dose) vs ARCO^®^ (2x/day divided doses)*. This study was conducted in Benin (Central Africa) in a child population with uncomplicated falciparum malaria. Children received number of tablets based on bodyweight as a single dose versus same dose divided into two doses give 12 h apart. The therapeutic responses were monitored for 28 days. Therapeutic efficacy and safety were similar for both therapeutic dose regimens (*i.e.* both regimens were equally effective).

(vii) *ARCO^®^ alone*. Two studies were conducted in the adult populations with uncomplicated falciparum malaria: one in Nigeria and one in Myanmar [17], with no comparators. The therapeutic responses were monitored for 28 days. Both studies demonstrated high efficacy and safety profile for ARCO^®^ in the respective country settings. The above information is summarized in Table 1.

**Table 1:** Summary data of different studies in different countries (CR = cure rates;ACPR = adequate clinical and parasitological response; PCT = parasite clearance time; FCT = fever clearance time).

Countries	Study design (ARCO® *vs.* others)	Sample size	Measured outcomes		
			CR/ACPR (%)	PCT (h)	FCT (h)
Papua New Guinea (adults)	ARCO®	51	100	24	19.8
	CQ + SP	49	98	40	32.0
Indonesia (adults)	ARCO®	78	99	28	13
	DHA + PPQ	74	97	26	11
Myanmar (adults)	ARCO®	53	98	21	18
	No comparator		__	21	18.2
Sudan (adults)	ARCO®	129	98	28	12
	Art/SP	71	97	__	14
	Art	65	95	__	11
Uganda (adults)	ARCO®	118	100	27	24
	AL	116	95	__	__
Uganda (children)	ARCO®	113	98	__	__
	AL	112	98	__	__
Nigeria (adults	ARCO®	74	92	24	19.5
	No comparator		__	__	__
Nigeria (children)	ARCO®	50	100	32	24.7
	AL	46	96	__	__
Nigeria (mixed)	ARCO®	62	100	24	__
	AL	58	96	48	__
	Art/AQ	54	100	24	__
Benin (children)	ARCO® (1-dose)	25	96	24	36
	ARCO® (2÷doses)	25	96	__	24
Ivory Coast (mixed)	ARCO®	60	100	25	24
	AL	60	98	__	24

Because the methodology of clinical and/or therapeutic assessment had not been prospectively standardized prior to the conduct of the studies, there existed substantial inter-study differences in defining, assessing, reporting, and classifying efficacy and adverse events. However, all studies were conducted in accordance with the World Health Organization (WHO) guidelines for antimalarial drug efficacy assessment [30]. 

### 3.2. General Review Findings

#### 3.2.1. Therapeutic efficacy

Analysis of the pooled data did not show any significant differences between ARCO^®^ treatment and other artemisinin-based combinations (artemether-lumefantrine, dihydroartemisinin-piperaquine, and artemether-SP) in the rate of reduction of parasite biomass in the blood, except for the non-artemisinin-based combination [19]. All artemisinin-based combinations reduced the parasite biomass by greater than 95% at 24/48 h across a wide range of baseline parasitaemias (range: 1,000-200,000 parasites/µL) from different study sites (data not shown). However, for ARCO^®^, it would seem remarkable for a drug combination with shorter duration of treatment within the family of ACTs. With the exception of few cases, in all the studies fever was cleared within 24 h following treatment (Table 1). In studies where gametocytes were measured or noted, the ARCO^®^ and other artemisinin-based combinations had a negative impact on the gametocytogenesis. 

All the studies demonstrated that ARCO^®^, artemether-lumefantrine, artesunate-amodiaquine, and dihydroartemisinin-piperaquine combinations were equally effective in the treatment of uncomplicated malaria, particularly *Plasmodium falciparum* malaria, while further studies are needed for *Plasmodium vivax* and mixed parasite species infections. One study showed low response with ARCO^®^ in the treatment of mixed infections, an observation needing further verification. The pooled data however, demonstrated that a single dose administration of ARCO^®^ is highly efficacious, both in adults and children. It was also noted that all patients responded satisfactorily to the shorter (a single dose or 1-day treatment) course of ARCO^®^. The cure rates however, were similar in all treatments on day-28; extension of observations beyond 28 days did not result in better outcomes nor was there any significant differences in cure rates whether ARCO^®^ was given as a single dose (8 tablets for adults or on a body-weight basis for children) or in two divided doses over 24 h (1-day treatment regime).

#### 3.2.2. Safety profile

The safety data provided in relation to individual patients were primarily clinical. Where laboratory data was available, the laboratory evaluation schedules were not consistent between the studies, making comparative interpretation of safety data difficult. Because the trial methodology included in the pooled analysis had not been prospectively standardized, there existed substantial inter-trial differences in defining, assessing, reporting, and classifying adverse events. Furthermore, reliably distinguishing drug side effects from clinical symptoms of malaria infection is often difficult and much of the reporting is largely dependent upon a subjective assessment performed at the time of the event. The safety data obtained for this analysis were from individual patient data case record forms, which were archived at the Centre for clinical studies at Kunming Pharmaceutical Corporation. 

A total of 16 different adverse events, with varying frequencies and intensities, were recorded in the pooled analysis of 952 adult patients who received artemisinin-naphthoquine combination for uncomplicated malaria. The five most common adverse events were, in order of frequency, headache, nausea, vomiting, dizziness, and abdominal pain. However, it was difficult to discern which of the adverse events were malaria related and which were due to drug treatment because almost all of these events were reported during the first 24-hrs following the commencement of treatment. No adult or paediatric patients discontinued the treatment or any part of the treatment prematurely due to adverse experiences.

A meaningful comparison of safety profiles between different treatments including artemisinin-based products is beyond the scope of this analysis. It should be noted however, that most of the safety data presented were derived from patients treated with ARCO^®^ and extrapolation from this data to different populations and settings should be made with caution. Despite the methodological limitations of this analysis, the overall safety profile of ARCO^®^ treatment in these series of studies appeared to be benign. The total incidence of drug-related adverse events considered by clinicians and/or principal investigators was estimated to be low (≤5%). The majority of the adverse events reported has been of gastrointestinal-related in nature and were self-limiting. In general, safety profile of ARCO^®^ treatment appears to be excellent. However, the data from this analysis do not suggest that there are no additional concerns about ARCO^®^ use as a therapeutic agent in a wider context but pharmacovigilance is important for its continued deployment. 

### 3.3. Discussion

Artemisinin-naphthoquine combination (ARCO^®^) is a new generation ACT. An analysis of the pooled data from several studies in different countries indicates that this combination is quite safe and equally effective (efficacious) as other ACTs recommended by World Health Organization (WHO) which include artemether-lumefantrine (AL), artesunate-amodiaquine (AS + AQ), artesunate-mefloquine (AS + MQ), artesunate plus sulphadoxine-pyrimethamine (AS + SP) and dihydro-artemisinin-piperaquine (DHA + PPQ) combinations for treatment of uncomplicated falciparum malaria [31]. Whether or not a single dose or a 1-day treatment with ARCO^®^ will ever be considered for malaria treatment one day (from a drug resistance point of view) on a wide scale remains to be debated but these series of studies undertaken in different countries provide additional information on the potency and/or efficacy of ARCO^®^ in different environmental and epidemiological conditions/settings. These and other studies further affirms that ACTs including ARCO^®^, represent a promising approach to drug-resistant malaria treatment [31,32,33,34,35,36,37,38], and a shorter regimens may offer certain advantages in settings where acceptability or adherence to complex therapeutic dosing regimens is an on-going challenge. Treatment failures with ACTs due to lack of proper adherence to best practices or due to lack of poor knowledge with multiple dosing and complex regimens have been reported [4,39,40]. The declining efficacy of artemisinin (or perhaps insufficient dose of the drug) may affect the efficacy of partner medicines after long term utilization. Naphthoquine, the partner drug in ARCO^®^ easily develops resistance if given as single dose. Resistance to the artemisinin’s partner medicines may compromise the efficacy of the ACT. Therefore, the efficacy of ARCO^®^ needs careful monitoring after its introduction and widescale use, particularly in multidrug resistant areas. At present however, there is seemingly no doubt that a single dose or a 1-day treatment with the combined artemisinin and naphthoquine for uncomplicated falciparum malaria provided high efficacy in adult populations, as well as in the trialed children population. 

The safety of ARCO^®^ is comparable to other ACTs; it is relatively safe, both from the laboratory and clinical aspects. The naphthoquine component may have some effects on certain hepatic functions but a review of data in a number of studies that assessed laboratory indices did not reveal any major deviations in hepatic functions that may implicate exclusively drug-induced hepatic dysfunctions [19]. Almost all biological indices returned to within normal range values post-treatment. Many of the reported post-treatment events were likely to be related to the underlying malaria infection rather than the drug(s) used in the treatment. Abnormalities in laboratory test reported in the therapeutic trials were limited to elevations of transaminases and frequency of abnormalities varied across studies treated with ACTs including ARCO^®^. No patients were intentionally discontinued prematurely as a result of severe adverse experiences during the therapy and monitoring periods, however, there were patients who withdrew or failed to fulfill the study protocols who remained to be accounted for in several of the studies. 

The pooled data from multi-national therapeutic studies presented here provides scientific evidence for clinical use of ARCO^®^ (artemisinin-naphthoquine combination) as a new generation ACT. The findings are consistent with observations made with other ACTs [32,33,34,35,36,37,38]. In general, malaria therapy with artemisinin-based combinations has been shown to reduce malaria transmission, as well as reducing malaria-related morbidity and mortality [41,42,43]. The reduction of malaria transmission may be attributed partly to the anti-gametocytocidal effects of artemisinin component of artemisinin-based combinations. Rapid intervention with artemisinin-based combination treatment in uncomplicated or severe malaria can also impact on mortality, particularly among children. This intervention would likely to result in survival benefit and better outcome. Shorter therapeutic dosing regimens such as offered by ARCO^®^, may be an advantage in that the health workers can administer the drug as directly observed treatment. With the shorter treatment duration, the potential to be deployed for home management of malaria at community levels needs to be evaluated. However, the impact of shorter dosing regimens on morbidity and mortality due to falciparum malaria remains to be ascertained.

Only one study in these series compared the efficacy of a single dose ARCO^®^ versus multiple doses DHA-PPQ on vivax malaria; both treatments provided equivalent cure rates (99% *vs.* 97% for ARCO^®^ and DHA-PPQ, respectively) on 42-day follow-up. Data is limited on the role of ARCO^®^ in other malaria parasite species including *Plasmodium vivax* malaria. 

## 4. Conclusions

Artemisinin-naphthoquine combination (ARCO^®^) compares favourably with existing ACTs, so may be the next new generation ACT. Review of available data concludes that, from the clinical efficacy and safety point of view, ARCO^®^ offers no advantages over existing ACTs except for the fact that this combination may be given as a single dose than three daily doses or three twice daily doses. It will be more useful particularly in situations where noncompliance to the currently practiced 3-day course of ACTs is common. 

## 5. Identified Research Gaps

While the therapeutic assessment of ARCO^®^ demonstrated high level of efficaciousness and safety, the following gaps in our knowledge exist.

### 5.1. Children

Malaria affects children more than adults. There is however, insufficient information available on the pharmacokinetics of ARCO^®^ in children, particularly in children between 6 months to 5 years of age. 

### 5.2. Pregnancy

Women who are pregnant are relatively more vulnerable to malaria than non-pregnant women. Denying this population the most effective antimalarial drugs available would not save lives. It may be that the embryonic toxicity of ACTs in human pregnancies has been over-emphasized [44]. There is therefore, an immediate need to establish safety perimeters within which ACTs including ARCO^®^ can safely be prescribed in pregnancies, particularly in the first trimester of pregnancy. 

### 5.3. Therapeutic Dosing Regimen and Pharmacokinetics

ARCO^®^ tablets were co-formulated to deliver optimal blood levels for individual drugs in a single dose administration of eight tablets. Any variations, such as two divided doses (1-day treatment) or scaling-down of number of tablets on body-weight basis must be supported and guided by pharmacokinetic data to avoid sub-therapeutic levels. Substantially little pharmacokinetic data is available to support and guide different dosage schedules, so further studies are needed in these areas to optimize therapy for different dosing schedules.
